# Une toxidermie mimant un lupus érythémateux aigüe chimio-induit

**DOI:** 10.11604/pamj.2018.29.66.6083

**Published:** 2018-01-24

**Authors:** Saoussane Kharmoum, Meryem Soughi

**Affiliations:** 1 Université Mohamed V, Suissi, Faculté de Médecine et de Pharmacie de Rabat, Service d’Oncologie Médicale, Centre Régional d’Oncologie de Tanger, Maroc; 2 Service de Dermatologie, Centre Hospitalier Régional Mohamed V, Al Houceima, Maroc

**Keywords:** Toxidermie, chimiothérapie, lupus erythémateux aigue, Toxidermia, chemotherapy, acute lupus erythematosus

## Abstract

Acute chemotherapy-induced lupus erythematosus (ALE) is rare. A few cases have been reported in the literature criminalizing capecitabine, paclitaxel and docetaxel. We report the case of a 64-year old female patient without a history of autoimmune diseases or of drug allergy followed up for invasive ductal carcinoma in the right breast immediately metastatized to the liver and to the lymph nodes. After AC60 first line chemotherapy regimen (a total of 6 cycles), she was treated with docetaxel at a dose of 100 mg/m^2^. After 5 cycles, she had diffuse erythematous lesions on both hands, forearms, cheeks and on the peribuccal area. She underwent corticosteroid therapy with sun protection and could continue the same chemotherapy until the eighth cycle. Patient’s evolution was marked by the progression of the disease. She was treated with capecitabine at a dose of 1250 mg/m^2^ twice a day. After six cycles she had erythematosquamous and itchy patches on the face resembling the wings of a butterfly (Panel A, Panel B) with oral ulceration and digital pulpitis (Panel C). This initially suggested acute chemotherapy-induced cutaneous lupus erythematosus. Biopsy suggested lichenoid toxidermia. Immunological assessment was performed to exclude chemotherapy-induced cutaneous lupus erythematosus, which showed anti-native DNA antibodies and negative anti-histone antibodies. Anti-nuclear antibody test is positive at 320; this test may be positive in 50-70% of patients with breast cancer, ENT or lymphoma. In the light of these results the diagnosis of toxidermia was the more likely.

## Image en médecine

Le Lupus érythémateux aigue(LEA) chimio-induit est une affection rare, quelques cas ont été rapporté dans la littérature incriminant la capecitabine, le paclitaxel et le docetaxel. Nous rapportons le cas d’une patiente âgée de 64 ans suivie pour carcinome canalaire invasif du sein droit, d’emblé métastatique au niveau hépatique et ganglionnaire, sans antécédents de maladies auto-immunes ou d’allergie médicamenteuse. Après une première ligne de chimiothérapie type AC60 (6 cures au total), elle a reçu le docetaxel à la dose de 100 mg/m^2^, après 5 cures elle a présenté des lésions érythémateuses diffuses au niveaux des deux mains, des avants bras, des deux joues et en péribuccale, elle a été mise sous corticothérapie avec protection solaire, on a pu poursuivre la même chimiothérapie jusqu’à la huitième cure. L’évolution a été marqué par la progression de sa maladie elle a été mise sous capecitabine à la dose de 1250 mg/m^2^ deux fois par jours, après six cures elle a présenté des plaques érythémateux-squameuse et prurigineuse du visage, disposée en aille de papillon avec ulcération buccale et pulpite des doigts (Panel C) faisant évoqué en premier un lupus érythémateux cutané aigue chimio-induit. Une biopsie a été réalisée qui était en faveur d’une toxidermie lichénoide. Un bilan immunologique a été demandé pour éliminer un LEA chimio-induit, objectivant des anticorps anti DNA natif et des anticorps anti-histones négatifs, les anticorps anti-nucléaires sont positives à 320, ces derniers peuvent être positives dans 50 à 70% des cas de cancer mammaire, ORL ou lymphome. Au vu de ces résultats le diagnostic de toxidermie est le plus probable.

**Figure 1:**
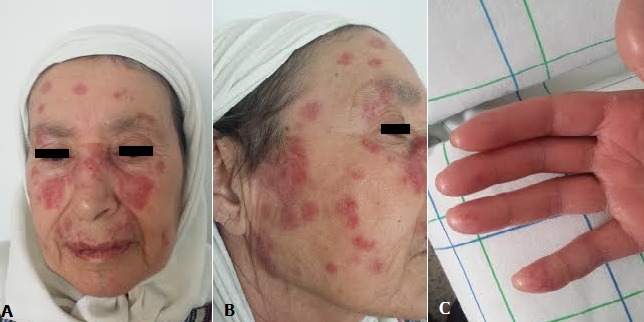
**A**) plaques érythémateux-squameuse du visage, disposée en aille de papillon; **B**) plaques érythémateux-squameuse du visage; **C**) pulpite des doigts

